# Pulmonary Nocardiosis in the Immunocompetent Host: Case Series

**DOI:** 10.1155/2015/314831

**Published:** 2015-09-30

**Authors:** Inderjit Singh, Frances Mae West, Abraham Sanders, Barry Hartman, Dana Zappetti

**Affiliations:** ^1^Department of Medicine, Division of Pulmonary and Critical Care, Weill Cornell Medical College, New York, NY 10065, USA; ^2^Department of Medicine, Division of Infectious Diseases, Weill Cornell Medical College, New York, NY 10065, USA

## Abstract

Pulmonary nocardiosis is commonly recognized as an opportunistic infection in patients with predisposing immunosuppressive conditions. However, reports of pulmonary nocardiosis in the immunocompetent host are rare. Here, we report a case series of four patients with pulmonary nocardiosis without a predisposing condition.

## 1. Introduction

Nocardiosis is caused by Gram-positive, weakly acid-fast, filamentous aerobic actinomycetes.* Nocardia* species are ubiquitous in our environment. Human infection occurs from direct inoculation of skin or soft tissues or via direct inhalation of* Nocardia* species [1]. Nocardiosis is more likely to affect immunosuppressed patients particularly those with depressed cell-mediated immunity [2]. However, approximately one-third of patients with nocardiosis do not have a predisposing immunosuppressive condition [3].

## 2. Case 1

A 71-year-old female with chronic obstructive pulmonary disease (COPD) (Forced Expiratory Volume in one second of 29% predicted) presented with a one-month history of progressive exertional dyspnea, productive cough, and wheezing. Over the past 6 months, she received five courses of oral prednisone for recurrent COPD exacerbations. One week before admission, she received a 10-day course of Levofloxacin after her sputum culture grew pan-sensitive* Pseudomonas aeruginosa*. She was an ex-smoker of 105 pack-years.

**Figure 1:**
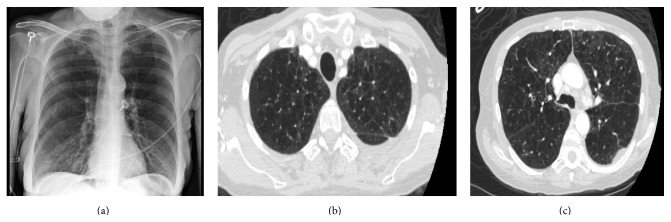
Chest X-ray and chest CT findings in Case 1. Chest X-ray findings on day of admission demonstrating hyperinflated lung fields. Chest CT findings 10 months before case presentation demonstrating severe centrilobular emphysema.

On admission, her temperature was 36.5°C, blood pressure 153/68 mmHg, oxygen saturation on 2 liters 99%, pulse rate 110 beats/min, and respiratory rate 22 breaths/min. On respiratory exam, she had diffuse wheezing. Chest X-ray demonstrated hyperinflated lungs fields (Figure 1). Chest computed tomography (CT) performed 10 months earlier demonstrated severe centrilobular emphysema (Figure 1). She had leukocytosis on admission (13.3 × 10^3^/*μ*L and 96% neutrophils).

Her admission sputum culture grew pan-sensitive* Pseudomonas aeruginosa* again. However, she continued to have ongoing wheezing and productive cough. Eventually,* Nocardia cyriacigeorgica* was identified on her acid-fast bacilli (AFB) sputum culture. She was started on Trimethoprim/Sulfamethoxazole (800/160 mg one tablet twice daily). Over the course of the next twelve months, she had repeated in-patient admission for worsening respiratory status. Subsequent sputum cultures repeatedly grew carbapenem-resistant* Acinetobacter baumannii. Nocardia* was never isolated again. She eventually died on home hospice care.

## 3. Case 2

A 68-year-old female with a history of left breast adenocarcinoma presented with a two-year history of productive cough and increasing dyspnea. She is an ex-smoker of 30 pack-years. She was diagnosed with breast cancer three years ago and underwent chemotherapy (Doxorubicin and Cyclophosphamide followed by Paclitaxel) followed by mastectomy and radiation therapy.

**Figure 2:**
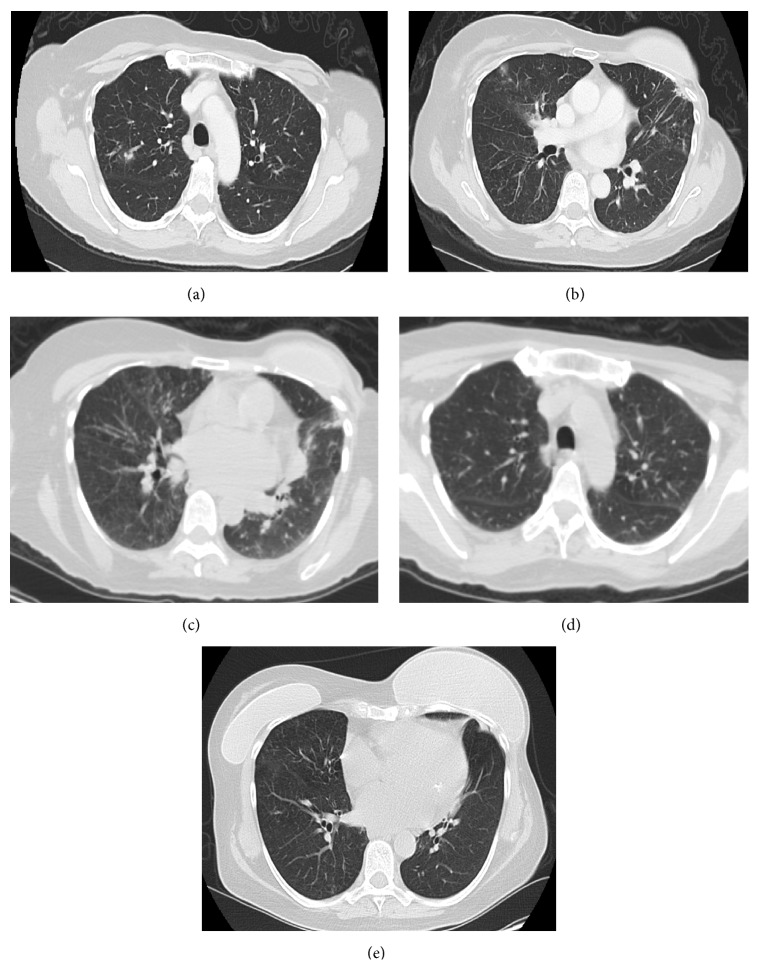
Chest CT findings in Case 2. Right upper and middle lobes nodular opacities (a) and lingular bronchiectasis (b). Waxing and waning opacities involving the right upper and middle lobes and lingula (c). Interval resolution of right middle lobe (d) and lingular opacities (e).

Her chest CT showed bronchiectasis and right sided tree-in-bud opacities (Figures 2(a) and 2(b)). The leading diagnosis at this stage was* Mycobacterium avium-intracellulare* complex (MAC) related lung disease. She was given a 14-day course of empiric Levofloxacin for treatment of bronchiectasis. Repeat chest CT eight weeks later showed waxing and waning parenchymal opacities with improvement in the right upper lobe but new nodular opacities involving the right middle lobe and lingula (Figure 2(c)). However, she continued to experience intermittent fevers with associated productive cough. Her sputum was sent for Gram stain and cultures grew* Nocardia cyriacigeorgica*.

She was treated with Trimethoprim/Sulfamethoxazole (800/160 mg DS twice daily). She improved clinically but developed urticarial rash. She was switched to inhaled Tobramycin (300 mg twice daily for 8 weeks) and oral Linezolid (600 mg twice daily). She improved clinically with radiographic resolution of disease (Figures 2(d) and 2(e)).

## 4. Case 3

A 75-year-old woman with COPD (Forced Expiratory Volume in one second of 32% predicted) and chronic MAC colonization with bronchiectasis presented with two-month history of progressive exertional dyspnea, productive cough, and constitutional symptoms. Several days before her presentation, she was started empirically on Amoxicillin/Clavulanate. An expectorated sputum sample grew MAC. Notably, in the past, a routine sputum culture grew* Nocardia cyriacigeorgica*; however, she was not treated due to stable symptoms. She presented to the emergency room because her symptoms persisted.

**Figure 3:**
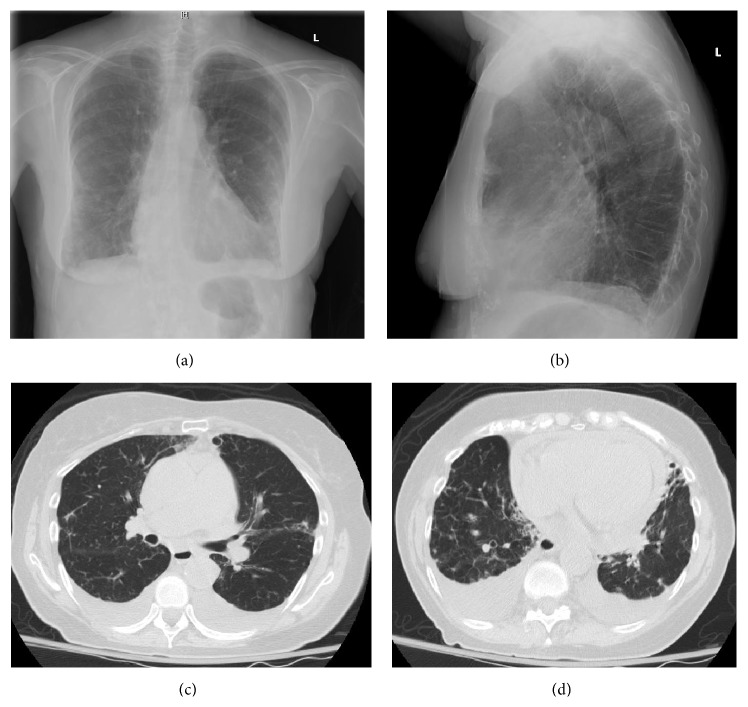
Chest X-ray and chest CT findings in Case 3. Chest X-ray showing stable bilateral nodular opacities and lower lobe predominant interstitial reticular thickening with new small bilateral pleural effusions (a, b). CT of the chest reveals bronchiectasis in the right middle and lingular lobes (c) and tree-in-bud nodular infiltrates in the bilateral lower lobes with new bilateral moderate pleural effusions.

On initial evaluation, she was afebrile with right basilar rhonchi. Laboratory investigations were notable for leukocytosis (12 × 10^3^/*μ*L and 79% neutrophils). Her chest X-ray showed stable bilateral nodular opacities (Figures 3(a) and 3(b)). She was treated empirically with Trimethoprim/Sulfamethoxazole for the previously identified* Nocardia cyriacigeorgica*. Chest CT showed bronchiectasis with bilateral lower lobe tree-in-bud infiltrates (Figures 3(c) and 3(d)). She underwent bronchoscopy with lavage which revealed no growth and bronchial wash that grew MAC. Her antibiotics were discontinued. She was treated with diuretics for worsening pulmonary hypertension. She clinically improved.

Following discharge, she developed worsening exertional dyspnea and cough. Sputum culture at that time grew* Nocardia cyriacigeorgica *and* Pseudomonas putida. *She was given Levofloxacin for treatment of the latter. However, she continued to complain of dyspnea and productive cough. Repeat echocardiogram showed improvement in her pulmonary hypertension. Her sputum culture again grew* Nocardia cyriacigeorgica*. She was then started on Trimethoprim/Sulfamethoxazole. During subsequent office visits, she reported subjective improvement in her respiratory status. She continued treatment with Trimethoprim/Sulfamethoxazole for eleven months and subsequent sputum cultures have not grown* Nocardia* spp.

## 5. Case 4

A 77-year-old woman with a history of bronchiectasis and MAC colonization presented with increasing sputum production. She is an ex-smoker with a 10-pack-year smoking history. She had a history of breast cancer treated with mastectomy and hormonal therapy. Three years ago, she underwent left lower lobectomy for lung adenocarcinoma. MAC infection was diagnosed on bronchoscopy eight years earlier and treated with triple drug therapy with symptom resolution. Subsequent sputum cultures revealed continued colonization with MAC. One week prior to presentation, she developed fever and cough and was prescribed Levofloxacin. Her fever resolved, but she continued to have persistent cough despite a short course of oral corticosteroid treatment.

**Figure 4:**
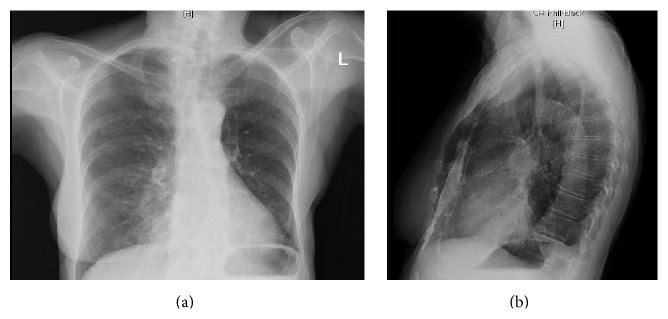
Chest X-ray findings in Case 4. Chest X-ray shows prior left lower lobectomy, stable bilateral nodules, and new consolidation versus atelectasis of the lingula.

On presentation, she was afebrile with room air oxygen saturation of 97%. Her chest radiograph is shown in Figure 4. An expectorated sputum sample was obtained for culture. The sputum culture grew multiple organisms, including* Pseudomonas aeruginosa*,* Staphylococcus aureus*,* Chryseobacterium indologenes,* MAC, and* Nocardia nova*. She was treated for bronchiectasis exacerbation with Levofloxacin, which relieved her fever and cough. She is being actively monitored for pulmonary nocardiosis with symptom monitoring and repeat sputum cultures. She has remained asymptomatic and has not been treated for* Nocardia* so far.

## 6. Discussion

Pulmonary nocardiosis is the most common manifestation of* Nocardia* infection. The estimated annual incidence of pulmonary nocardiosis in the United States is between 500 and 1000 cases [4]. The majority of patients have impaired cell-mediated immunity, including those with underlying malignancies and human immunodeficiency virus infection, solid-organ or hematopoietic stem cell transplant recipients, and those receiving long term corticosteroids and medications that suppress cell-mediated immunity [1]. Preexisting pulmonary disorders such as COPD [5], bronchiectasis [6], and MAC lung disease [6] are additional risk factors. In this patient population, an intrinsic defect in airway clearance and bacterial colonization of the lower airways alter ciliary motility and hasten epithelial destruction, facilitating nocardial infection. Most cases of pulmonary nocardiosis in COPD patients occur with concurrent corticosteroid use [5, 7, 8].  Table 2 highlights the predisposing conditions that may have contributed to pulmonary nocardiosis in our patients.

Clinical and radiographic features of pulmonary nocardiosis often resemble those of fungal and mycobacterial disease [8]. The identification of* Nocardia* from asymptomatic immunocompetent patients should be interpreted cautiously. The clinical status of the patient in Case 4 is being closely monitored; the decision of treatment will depend on the development of symptomatic disease. The patient in Case 4 represents* Nocardia* colonization rather than infection because her presentation of fevers and productive cough resolved following institution of Levofloxacin. Following her treatment course, she continues to remain asymptomatic. In Case 2, the constitutional symptoms and chest CT features of nodular opacities and bronchiectasis resemble MAC lung disease; thus clinicians often overlook the less common pulmonary nocardiosis. Taken together, the protracted incubation period along with its nonspecific presentation and radiographic features makes the diagnosis of pulmonary nocardiosis challenging.

The diagnosis of pulmonary nocardiosis requires isolation and identification of the organism in respiratory secretions. Sputum cultures are positive in approximately 90% of patients. If patients are unable to expectorate, bronchoscopy with bronchoalveolar lavage can be considered. The diagnostic yield with bronchoalveolar lavage was reported as 100 percent in one study [9]. However, in Case 3, the bronchoalveolar lavage specimen was not diagnostic. Staining with modified acid-fast and Gram stains allows for a presumptive diagnosis to be made. Currently,* Nocardia* genus has over 50 species that have been characterized using various phenotypic and molecular techniques [8]. Molecular techniques such as polymerase chain reaction (PCR) testing, restriction enzyme analysis, and 16s ribosomal-DNA sequencing have enhanced the identification of the different* Nocardia* spp.* Nocardia* species in all cases were isolated using the 16s ribosomal-DNA sequencing technique.

Accurate* Nocardia* spp. identification is important because of the variation in antibiotic susceptibilities seen between the various* Nocardia* spp. (Table 1) [10]. In vitro susceptibility testing should therefore complement the identification of* Nocardia* isolates from clinical specimens.

The treatment of choice for pulmonary nocardiosis is Trimethoprim/Sulfamethoxazole (divided doses of 5 to 10 mg/kg per day of the Trimethoprim component are recommended). Alternative agents include Amikacin, Imipenem, Meropenem, Ceftriaxone, Cefotaxime, Minocycline, Moxifloxacin, Levofloxacin, Linezolid, Tigecycline, Amoxicillin/Clavulanate, and Tobramycin. The duration of treatment varies and depends on patient's clinical response because susceptibility testing does not always correlate with clinical outcome. In a prior case series of patient with nocardiosis, a favorable response was reported in 89% of patients with susceptible strains and in 75% with Trimethoprim/Sulfamethoxazole-resistant strains [11]. Along with emerging Trimethoprim/Sulfamethoxazole-resistant strains, it is reasonable to consider combination therapy and alternative agents with a proven clinical response to treat nocardiosis [12]. Immunocompetent patients with pulmonary nocardiosis are treated for at least six to twelve months [1]. There have not been any randomized controlled trials to determine the optimal agent(s), route of administration, or treatment duration for patients with pulmonary nocardiosis.

The mortality rate from pulmonary nocardiosis in immunosuppressed patients is approximately 40% [13, 14]. This increases to 64% in those with disseminated disease and 100% in those with central nervous system (CNS) involvement [13]. Patients with pulmonary nocardiosis should therefore be considered for brain imaging and blood culture testing to assess possible disseminated or CNS disease.

## 7. Conclusion

This case series highlights the notion that pulmonary nocardiosis should be included in the differential diagnosis even among immunocompetent patients. It is important to recognize the predisposing factors in this patient group and to differentiate nocardial infection from colonization. A high index of clinical suspicion together with close collaboration with the microbiology laboratory allows for more accurate diagnosis in order to initiate appropriate therapy with the purpose of reducing mortality.

## Figures and Tables

**Table 1:** Antimicrobial susceptibilities of *Nocardia* species isolated from sputum cultures.

Antibiotic	*Nocardia cyriacigeorgica*			*Nocardia nova*
	Case 1 MIC	Case 2 MIC	Case 3 MIC	Case 4 MIC
	(*μ*g/mL)	(*μ*g/mL)	(*μ*g/mL)	(*μ*g/mL)
Amikacin	≤8 S	≤1	≤1 S	≤8 S
Amoxicillin/Clavulanate	16/8 Int.	32/16 R	4/2 S	>32/16 R
Azithromycin	64 Int.	Not available	Not available	≤16 S
Cefepime	8 S	Not available	Not available	≤4 S
Cefotaxime	≤8 S	Not available	Not available	≤8 S
Ceftriaxone	≤8 S	8 S	≤4	≤8 S
Ciprofloxacin	>8 R	4 R	>4 R	>8 R
Clarithromycin	8 Int.	>16 R	8 R	≤25 R
Clofazimine	≤0.5 S	Not available	Not available	0.5 S
Doxycycline	Not available	4 Int.	8 R	Not available
Gentamicin	≤2 S	Not available	Not available	16 R
Imipenem	≤2 S	4 S	≤2 S	≤2 S
Kanamycin	16 R	Not available	Not available	16 R
Linezolid	2 S	2 S	2 S	≤1 S
Minocycline	≤1 S	2 Int.	>8 R	4 S
Moxifloxacin	Not available	4 R	4 R	Not available
Tobramycin	≤2 S	≤1 S	≤1 S	>16 R
Trimethoprim/Sulfamethoxazole	≤0.5/9.5 S	≤0.25/4.8 S	0.5/9.5 S	2/38 S

MIC: minimum inhibitory concentration; S: sensitive; R: resistant; Int: intermediate.

**Table 2:** Patient demographics, predisposing condition, and treatment course.

	Case 1	Case 2	Case 3	Case 4
Age (years)	73	69	79	78

Sex	Female	Female	Female	Female

Predisposing condition	COPD and concurrent steroid use	Bronchiectasis	COPD and MAC colonization with bronchiectasis	MAC colonization

Pathogen	*Nocardia cyriacigeorgica*	*Nocardia cyriacigeorgica*	*Nocardia cyriacigeorgica*	*Nocardia nova*

Treatment	Trimethoprim/ Sulfamethoxazole	Inhaled Tobramycin and oral Linezolid	Trimethoprim/ Sulfamethoxazole	Observation

Duration	6 months	8 weeks	11 months	Continued observation

COPD: chronic obstructive pulmonary disease; MAC: *Mycobacterium avium-intracellulare* complex.
